# Non-pharmacological treatment of gambling disorder: a systematic review of randomized controlled trials

**DOI:** 10.1186/s12888-021-03097-2

**Published:** 2021-02-17

**Authors:** Eliana O. Ribeiro, Nuno H. Afonso, Pedro Morgado

**Affiliations:** 1 School of Medicine, University of Minho, Braga, Portugal; 2 Life and Health Sciences Research Institute (ICVS), School of Medicine, University of Minho, 4710-057 Braga, Portugal; 3 ICVS-3Bs PT Government Associate Laboratory, School of Medicine, University of Minho, 4710-057 Braga, Guimarães Portugal; 4 Hospital de Braga, Sete Fontes — São Victor, 4710-243 Braga, Portugal

**Keywords:** Gambling disorder, Systematic review, Treatment, Psychotherapy, Non-pharmacological therapy

## Abstract

**Background:**

The main focus of the non-pharmacological treatment of Gambling Disorder (GD) is the behaviour, cognition and motivation of the patient, addressing the psychological determinants of gambling. Although there is not a gold standard non-pharmacological treatment yet, many studies already had promising results, and the outcomes were even better when pharmacotherapies were combined with psychotherapies. This review intended to synthesise the efficacy of various available non-pharmacological therapies for GD evaluated in randomized controlled trials.

**Methods:**

A systematic search was conducted in PubMed and in Cochrane Library for randomized controlled trials. Studies were included if participants had GD as their primary diagnosis and excluded if patients had other comorbidities.

**Results:**

From 320 records identified, 22 studies were included in the critical appraisal. They included a total of 1694 patients, with a mean age of 42.94 years, and a 62.31% of males. Seven trials revealed the efficacy of cognitive behaviour therapy in improving significantly the outcomes. Three studies assessing cognitive therapy showed significant improvements in gambling symptoms, while one study showed improvements in gambling behaviour using exposure therapy. Combined or separate motivational interviewing and imaginal desensitization had significant results in 4 trials. Four other studies also showed efficacy for: couples therapy, node-link mapping therapy, 12-step facilitated and personalized feedback intervention. Physical exercise had promising results but did not reach significance.

**Conclusion:**

The literature included in this review showed the heterogeneity of available psychotherapies. The majority of studies supported the efficacy of the tested therapies, while some of them, due to limitations such as small sample sizes or inadequate control groups, failed to reach significance.

## Background

Gambling disorder (GD) is the term used to define a persistent and recurrent pattern of gambling that is associated with substantial distress or impairment, according to the fifth edition of the Diagnostic and Statistical Manual (DSM-5) [1]. The symptoms associated with this disorder are very similar to those seen in addictive disorders. Thus, in the DSM-5, GD is now classified as an addictive disorder rather than as an impulse control disorder (in DSM-IV) [2]. As Shaffer and Korn observed, patients with disorders such as kleptomania or pyromania (impulse control disorders) feel overwhelmed by their impulses to act and then, feel a sense of relief after having acted. In contrast, patients with GD find their gambling pleasant and only when they stop gambling or suffer losses, they begin to feel distress, as it happens in substance use disorders [3]. In fact, GD is now the first and only formal behavioural addiction in the DSM-5 [2]. Although any pharmacological treatment has yet to be approved for GD, many studies have already had promising results regarding its efficacy, both in pharmacological-only schemes and in combination with psychotherapies [4]. When combined, better rates of patient retention were found, in comparison to pharmacotherapy-only treatments [4]. Also, Topiramate combined with behavioural therapy could be used to treat patients without comorbidities, while Escitalopram may be effective for the treatment of patients with co-occurring affective disorders [2].

While the pharmacological treatment addresses the dysregulation in neurotransmitter systems, non-pharmacological treatments have a different approach, with a main focus on the behaviour, cognition and motivation of the patients, addressing the psychological determinants of gambling [2]..

Cognitive-behavioural therapies (CBT) are the most commonly used to treat GD [2]. The cognitive component approaches the thoughts, attitudes, and beliefs of the patients, that are the root of the behavioural problems, like gambling cravings or urges [5]. The behavioural component’s objective is to identify external triggers, practising with the patients their response to those triggers, and to find and promote gambling alternatives [2]. The aim of cognitive therapies (CT) is to correct cognitive distortions and irrational thoughts. Examples of these are patients’ overconfidence in their ability to identify systems of winning, beliefs that some behaviours or rituals may help collect wins, and even some statistically wrong beliefs about the games [5]. The objective of exposure therapies (ET) with response prevention is to induce gambling desires and urges in the patients, by exposing them to a gambling environment or by giving them gambling cues, and then, to teach them how to resist those desires in a gradually more self-controlled way [6]. Motivational intervention therapies consist in a counselling approach focused on the person, helping them to explore and resolve ambiguities, what enhances the willingness to change behaviours [7]. The position of the interviewer is non-judgemental, non-confrontational and non-adversarial. Instead, it is collaborative, induces patients’ own motivation and honours their autonomy [2, 8]. This therapy’s intention is to motivate the patients to continue their treatment, considering that two-thirds of GD patients who seek treatment do not engage in it, giving up before it is completed [2]. The main objective of psychodynamic psychotherapy is to identify the meaning behind current behaviour and to resolve conflicts that may have led to it. Furthermore, this therapy focuses on reducing the guilt and shame associated with the consequences of pathological gambling [9]. Other types of therapies will also be reviewed in this systematic review, for example: self-help programmes based on the previous describe therapies; a systemic-based treatment, in this case, couples therapy, that focuses on the patients’ significant others, integrating them in the treatment process; a 12 step facilitated therapy, with the basic principles of gamblers anonymous; and physical activity, that has proved to be efficient in reducing depression and anxiety. Cowlishaw’s et al. [5] systematic review studied the efficacy of CBT, motivational interviewing, integrative therapies and other psychological therapies. Their results supported the efficacy of CBT in reducing gambling behaviour and symptoms, while motivational interviewing only reduced gambling behaviour. The authors also concluded that integrative therapies and other psychological therapies were beneficial, however, there were few studies and evidence to evaluate them. Gooding’s et al. [10] review concluded that cognitive therapy, motivational interviewing and imaginal desensitization had significant results. The authors also stated that when different types of therapy were compared, cognitive therapy was the more advantageous. In Petry’s et al. [11] review, it was concluded that although patients with less symptoms could benefit from minimal interventions, for patients with more severe gambling pathology some therapist contact may be necessary for them to benefit from the therapies.

The main objectives of this systematic review were to:

1. evaluate the efficacy and durability of treatment effects, in comparison with control conditions;
2. assess if there is a difference between short- and long-term treatments;
3. make a direct comparison between different therapies, and assess the benefits of combining some of them;
4. critically evaluate the main characteristics of some included studies that may have influenced part of the results and conclusions.

## Methods

### Search strategy

**Fig. 1:**
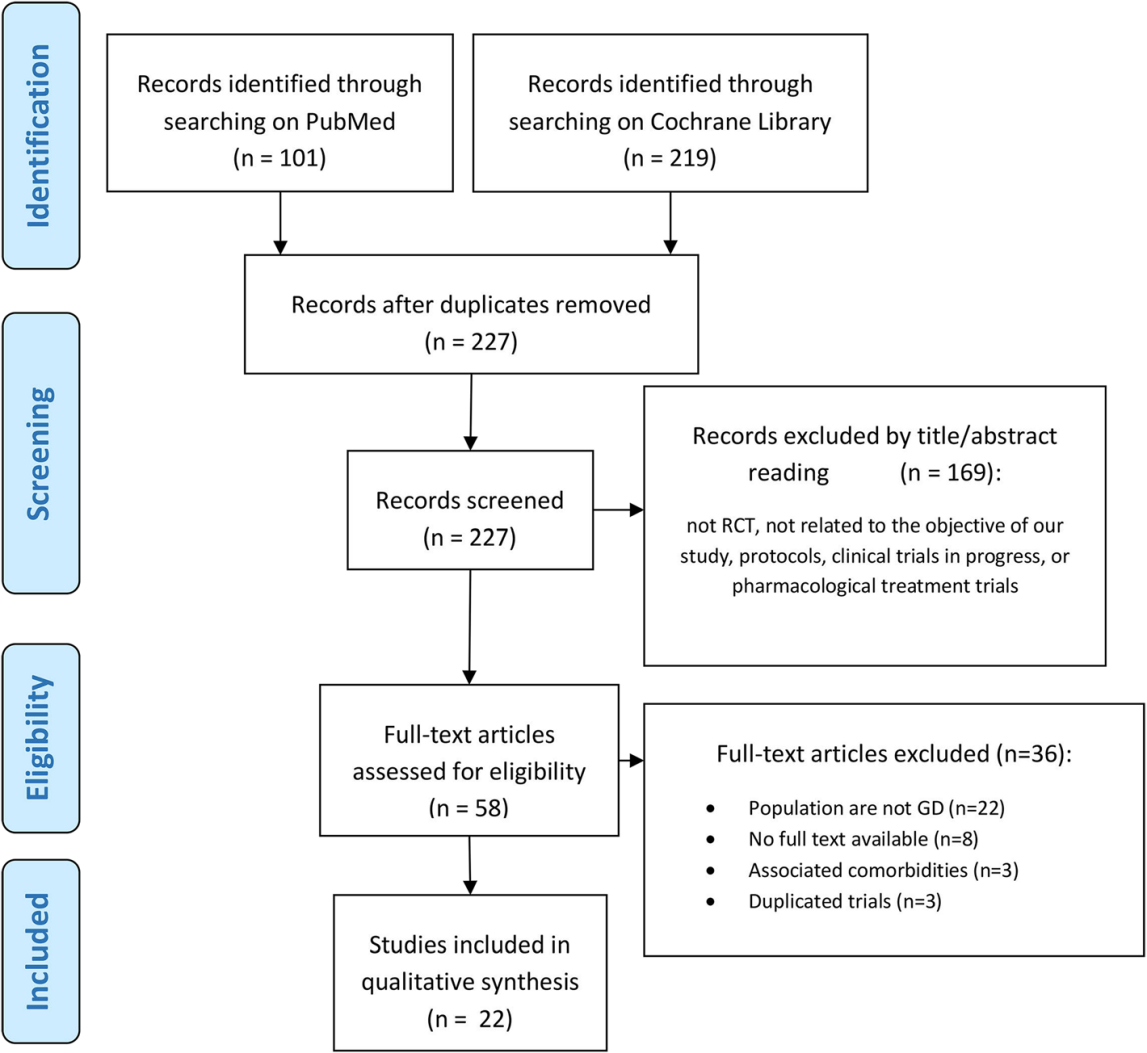
PRISMA flow diagram of the systematic review phases

The bibliographic searches were performed at PubMed and Cochrane Library databases. Searching only for randomized controlled trials performed in humans, published until February 29th 2020, the following strings were used: (gambling disorder or compulsive gambling or pathological gambling) AND (“cognitive–behaviour therapy” or “behaviour therapy” or CBT or “non-pharmacological treatment” or psychotherapy or “support group” or “gamblers anonymous” or “cognitive therapy” or “motivational therapy” or MET or “motivational intervention”). The study selection and screening process of identified studies is described in Fig. 1.

### Inclusion and exclusion criteria

We included only randomized controlled trials, written in English or Portuguese, performed in humans with a diagnostic of GD and related to non-pharmacological treatment of GD. Included participants could be of any age, ethnicity and sex. We excluded studies that required participants to have other comorbidities, and on those studies who used the same sample and evaluated the same treatment or outcomes, those with the largest sample and/or more time of follow-up were selected.

### Quality assessment in included studies

The included studies were assessed for quality according to the CASP checklist [12], specifically designed for randomized controlled trials. The aspects considered when appraising these trials answer four questions [12]: (1) is the basic study design valid for a randomized controlled trial?; (2) was the study methodologically sound?; (3) what are the results?; and (4) will the results help locally?

Three studies were assessed as having moderate quality, and the remaining were classified as having high quality.

### Measures

The outcome measures used through the studies were diverse. Detailed information about the scales and scores employed by the trials reviewed in this systematic review can be found in Table 1.

**Table 1:** Main characteristics of the scores and scales used as outcome measures

Outcome Measure	Description	Scale	Domain Measure
Beck Anxiety Inventory [13, 14]			
BAI	Self-report inventory that provides an accurate measure of anxiety, searching for symptoms of anxiety during the past week.	21 items scored on a scale value of 0 (not at all) to 3 (severely).	Measure of anxiety severity. Higher total scores indicate more severe anxiety symptoms (score ≥ 26 indicates severe anxiety).
Beck Depression Inventory II [14–16]			
BDI-II	Self-report instrument, based on DSM-IV, that provides a valid measure of depression, searching for how participants have been felling during the preceding 2 weeks.	21 items scored on a scale value of 0 (not at all) to 3 (severely).	Measure of depression severity (covering symptoms of depression, cognitions and physical symptoms). Higher total scores indicate more severe depressive symptoms (score ≥ 29 indicates severe depression).
Brief Symptom Inventory [17, 18]			
BSI	Self-report instrument which reflects the intensity of an individual’s mental health distress during the preceding week. Covers nine symptoms dimension (somatization, obsessive-compulsive, interpersonal sensitivity, depression, anxiety, hostility, phobic anxiety, paranoid ideation and psychoticism) and three global indices of distress: Global Severity Index, Positive Symptom Distress Index, and Positive Symptom Total.	53 items scored on a scale value of 0 (not at all) to 4 (extremely).	Measure psychological symptoms (symptomology, intensity of symptoms and number of reported symptoms). Higher total scores indicate more severe psychological symptoms.
+Canadian Problem Gambling Index [19–21]			
CPGI	Self-assessment instrument that measures problem gambling during the preceding year. Includes indicators of social and environmental context of gambling and problem gambling.	31 items, where 9 items are scored on a scale value of 0 (not at all) to 3 (severely).	Measures gambling severity. Higher total scores indicate more severe problem gambling (score ≥ 8 indicates problem gambler status).
Coopersmith Self-Esteem Inventory [16, 22]			
CSEI	Self-report questionnaire designed to measures the extent to which individuals customarily maintain a personal evaluation of competence, success, significance and worthiness.	50 items scored using a dichotomous scale (“like me” vs “unlike me”).	Measures specific aspects of self-esteem, namely, general self, social self-peers, home parents, and professional. CSEI scores can range from 0 to 50, with higher scores reflecting higher self-esteem.
Dyadic Adjustment Scale [18, 23]			
DAS	Self-administered questionnaire that measures the quality of marital relationships and can be used for any committed relationship.	32-items comprised of varying response scales.	Measures the relationship quality and comprises consensus, satisfaction, cohesion, and affectional expression. Higher total scores indicate less distress in relationship (score ≥ 114.8 indicates happily married couple).
Depression Anxiety Stress Scale [24, 25]			
DASS	Self-report scales that measure the negative emotional states of depression, anxiety and stress over the previous week.	42 items scored on a scale value of 0 (not at all) to 3 (severity/frequency).	Measure of depression, anxiety and stress. Higher total scores indicate more severe depression, anxiety or/and stress.
Diagnostic Interview for Gambling [20, 26]			
DIG	Individual diagnostic instrument for pathological gambling. Consists primarily of 20 multiple-choice questions, two addressing each of the DSM-IV criteria.	20 items, the total scored is assessed on a scale of 1–10.	Measures gambling severity. Higher total scores indicate more severe problem gambling (score ≥ 5 indicate pathological gambling status).
Gambling Follow-up Scale self-report version [27, 28]			
GFS-SR	Self-report questionnaire that evaluate gambling behaviour, impairments in social life and personal impairments in patients diagnosed with GD according to the DSM-5 criteria.	10 items scored on a scale value of 1 (severity) to 5 (not at all).	Assesses improvement in GD. Higher scores indicate greater improvement in gambling symptoms (scores ≥33 indicate recovery).
Gamblers Inventory of Negative Consequences [29]			
GINC	Self-report instrument to assess negative consequences of gambling during the previous 3 months, adapted from Drinker Inventory of Negative Consequences.	26 items rated on a scale 1 (never) to 7 (very often).	Measures negative consequences of gambling. Higher scores indicate more negative consequences of gambling.
Gambling Related Cognitions Scale [20, 25, 30]			
GRCS	Brief scale to screen the presence, nature and intensity of cognitive distortions among gamblers.	23 items rated on a scale 1 (strongly disagree) to 7 (strongly agree).	Evaluate gambling-related cognitive distortions. The higher the total score, the higher the number of gambling-related cognitions displayed.
The Gambling Symptom Assessment Scale [18, 25, 31]			
G-SAS	Self-report scale designed to assess gambling duration and urges, thoughts and preoccupations, control, emotional distress, and adverse personal consequences as a result of gambling.	12 items scored on a scale value of 0 (not at all) to 4 (extremely).	Measures gambling symptoms. Higher scores indicate more severe symptoms (scores ≥40 indicate extremely severe symptoms).
Gambling Urge Scale [32]			
GUS	Brief questionnaire based on the Alcohol Urge Questionnaire, measuring on a single factor the extent of gambling urge based on the participant’s self-reported thoughts and feelings.	6 items scored on a scale value of 0 (not at all) to 7(extremely).	Measures gambling urge. Higher scores indicate greater urges to gamble.
The Montgomery-Asberg Depression Rating Scale [33, 34]			
MADRS	Diagnostic instrument used to assess depression symptoms (sadness, inner tension, less sleep and appetite, concentration difficulty, lassitude, inability to feel, pessimistic and suicidal thoughts) in patients with mood affective disorders.	10 items rated on a scale 0 (not at all) to 6 (extremely).	Measure depression severity. Higher scores indicate more severe depression (scores ≥34 indicate severe depression).
National Opinion Research Center DSM Screen for Gambling Problems [35–37]			
NODS	Screening measure based on DSM-IV criteria for pathological gambling.	34 items scored from 0 to 10.	Assess gambling disorder. Scores of 5 or higher indicate gambling disorder.
Problem Gambling Severity Index [11]			
PGSI	Self-report measure of gambling behaviour over the previous 12 months.	9 items scored on a scale value of 0 (never) to 3 (always).	Measures gambling severity. Higher scores indicate more severe problem gambling. Scores of 8 or higher indicate gambling disorder.
Pathological Gambling – Yale Brown Obsessive-Compulsive Scale [38, 39]			
PG-YBOCS	Clinician administered scale that rates gambling symptoms within the previous 7 days, comprising an urge/thought subscale and a behaviour subscale.	10 items scored on a scale value of 0 (not at all) to 4 (extremely).	Measures gambling severity. Higher scores reflect greater illness severity.
South Oaks Gambling Screen [40, 41]			
SOGS	Reliable instrument for screening populations for gambling problems, based on DSM-III criteria.	20 items comprised of varying response scales.	Measure gambling severity. Higher scores reflect more severe gambling problems. Scores of 5 or higher indicate gambling disorder.
State-Trait Anxiety Inventory [16, 42]			
STAI	Self-report scales for measuring state and trait anxiety, for diagnose anxiety and to distinguish it from depressive syndromes.	40 items scored on a scale value of 0 (not at all) to 4 (very much so).	Measure anxiety. Higher scores indicate higher levels of anxiety.
Systemic Therapy Inventory of Change [43–45]			
STIC	Self-report instrument that assess individual, couple and family functioning and the alliance in family, couple, and individual therapy, in an integrative and multi-systemic perspective.	6 system scale with a total of 134 items comprised of varying response scales + 3 alliance scales	Measure relationship functioning. Higher scores represent worse adjustment.
The Gambling Timeline Followback [35]			
TLFB-G	Self-reported instrument that assess losses and days gambled in the previous 30 days, using the timeline followback methodology.	–	Measure gambling behaviour. Greater losses and days gambled indicate worse gambling behaviour.
Victorian Gambling Screen [46, 47]			
VGS	Three sub-scales of which the harm to self, others and the wider community is applied to determine problem gambling levels in the previous year.	15 items scored on a scale value of 0 (never) to 4 (always).	Measure gambling problems. Higher scores indicate more severe gambling problems. Score of 21 or higher indicates a gambling problem.

*DSM* Diagnostic and Statistical Manual of Mental Disorders, *GD* Gambling Disorder

Besides the scales summarized in Table 1, some studies also used other behavioural measures like money spent gambling per week, gambling frequency and time spent gambling per month.

## Results

### Description of studies

In Fig. 1 can be found the Preferred Reporting Items for Systematic reviews and Meta-analysis (PRISMA) flowchart with the results of our systematic search. The initial search retrieved 101 articles from PubMed database and 219 articles from Cochrane Library. Once duplicate and unrelated articles were removed, based on title and abstract, 58 full-text articles remained for further investigation. Later, another 36 articles were left out because the participants were not diagnosed with GD, were required to have comorbidities associated or the articles were not full-text. Finally, a total of 22 studies met the inclusion criteria for the current systematic review. All 22 studies included in this review were published between 1991 and 2019. Follow-up time ranged between 2 weeks and 9 years. Sample sizes ranged between 14 and 231 participants. A total of 1694 patients were included, from 8 different countries, with a mean age of 42.94. The percentage of males was 62.31%, and the percentage of caucasians ranged from 73 to 100%. Information about the inclusion and exclusion criteria can be seen in Table 2.

**Table 2:** Characteristics of the included studies and their population

Article	Country	Follow-up	Sample size	Demographics (mean age, gender, ethnicity)	Inclusion criteria	Exclusion criteria
Nilsson et al., 2019 [35]	Sweden	12 months	136	35.6 years 81.6% males	Scoring ≥5 in PGSI, live in Sweden, understand and write Swedish and be aged at least 18 years.	Severe psychiatric disorders.
Penna et al., 2018 [28]	Brazil	8 weeks	59	54.03 years 57.6% males 78% caucasians	Diagnostic according DSM-5 criteria, ability to understand the purpose of the study, physically able to engage in physical activity, for female patients a negative pregnancy test.	Severe psychiatric disorder or other medical condition requiring inpatient treatment.
Casey et al., 2017 [25]	Australia	12 months	174	44.37 years 40.98% males 79.62% caucasians	Diagnostic according DSM-5 criteria, over 18 years of age, reside in Australia	Receiving additional treatments, involved in legal proceedings, not proficient with English, at a high risk of suicide; were acutely psychotic, or if their gambling behaviour only occurred during manic episodes.
Bouchard et al., 2017 [20]	Canada	2 weeks	25	47 years 50% males	Diagnostic according DSM-5 criteria and be treated at Centre CASA or Maison Jean-Lapointe in Canada	━
Smith et al., 2015 [47]	Australia	9 months	99	46.49 years 49.43% males	Scoring ≥8 in SOGS, 18 years of age or older, gambling with electronic gaming machines, gambled in the past month	Being suicidal, exhibiting acute psychosis or mania or experiencing significant mental distress, psychological treatment for problem gambling in the previous 12 months
Lee et al., 2014 [18]	Canada	2 months	16	49.1 years 66% males 73% caucasians	One or both spouses diagnosed according DSM-IV criteria, gambled in the past 2 months, be at least 18 years of age and committed couple relationship	Suicidal ideation, attempt at suicide or psychotic symptoms for the past month, recurring intimate partner violence or receiving additional treatments
Grant et al., 2011 [38]	United States	6 months	68	49.01 years 66% males 94.12% caucasians	Diagnostic according DSM-IV criteria and gambled at least 1 time per week for the past 2 months	Past 3-month substance use disorder, positive urine drug screen, current pharmacotherapy or psychotherapy for GD, previous GA attendance, any clinically significant suicidal ideation or current use of psychotropic medications
Myrseth et al., 2011 [31]	Norway	6 months	30	32.8 years 86.67% males	Diagnostic according DSM-IV, minimum age of 18 years, not having used SSRIs for the last 6 months	Suffering from epilepsy or liver/kidney disorders, evidence of psychosis or mental disorders, alcohol or drug dependency
Marceaux et al., 2010 [48]	United States	6 months	49	46.57 years 34.69% males 85.71% caucasians	Diagnostic according DSM-IV and at least 21 years of age	━
Grant et al., 2009 [39]	United States	8 weeks	68	48.7 years 36.8% males	Diagnostic according DSM-IV and had gambled at least once per week for the past 2 months	Past 3-month substance use disorder, positive urine drug screen at screening, current psychotherapy or medication for GD, previous GA attendance or suicidal intentions
Myrseth et al., 2009 [29]	Canada	3 months	14	37.43 years 78.57% males	Diagnostic according DSM-IV, ≥5 in SOGS and over 18 years of age	Suffer from any type of substance abuse or from any psychotic disorder
Carlbring et al., 2009 [36]	Sweden	12 months	150	40.5 years 83.5% males	Scoring ≥5 on NODS, speak Swedish and ability to complete self-report questionnaires	Suicidal ideation, unwillingness to be randomized, medication for anxiety and/or depression, drug and/or alcohol dependence, or major mental disorders
Cunningham et al., 2009 [21]	Canada	3 months	49	44.41 years 48.11% males	Diagnostic according DSM-IV criteria and interested in participating in the study	━
Carlbring et al., 2008 [37]	Sweden	36 months	66	31.9 years 94% males	Scoring ≥5 on NODS, at least 18 years of age, live in Sweden and have gambled at least once in the past 30 days	Having > 21 on MADRS depression scale, > 4 on the suicide item or playing computer games without betting money
Dowling et al., 2007 [16]	Australia	6 months	56	43.58 years 100% females	Diagnostic according DSM-IV criteria	━
Petry et al., 2006 [41]	United States	12 months	231	44.87 years 54.98% males 84.42% caucasians	Diagnostic according DSM-IV criteria, gambled in the past 2 months, were 18 years or older, and ability to read	Current suicidal intentions, past-month psychotic symptoms, or already receiving gambling treatment
Melville et al., 2004 [14]	United States	6 months	19	52.58 years 15.79% males 89% caucasians	Diagnostic according DSM-IV criteria and SOGS	━
Ladouceur et al., 2003 [49]	Canada	24 months	71	43.41 years 77.97% males	Diagnostic according DSM-IV and willingness to undergo randomization	Evidence of current or past schizophrenia, bipolar disorder, or organic mental disorder
Ladouceur et al., 2001 [50]	Canada	12 months	101	41.98 years 82.81% males	Diagnostic according DSM-IV and be willing to undergo randomization	Evidence of immediate suicidal intent or current or past schizophrenia, bipolar disorder or organic mental disorder
Sylvain et al., 1997 [51]	Canada	12 months	29	40.19 years 100% males 100% caucasians	Diagnostic according DSM-III criteria, seeking help for gambling problems and rate motivation to change ≥7 (0–10)	━
Echeburua et al., 1996 [6]	Spain	12 months	64	35 years 44.44% males	Diagnostic according DSM-III criteria, ≥8 in SOGS and gamble primarily with slot machines	Suffering from other psychopathological disorders
McConaghy et al., 1991 [52]	Australia	2–9 years	120	42.53 years 90.83% males	Diagnostic according DSM-III criteria	Untreated active psychosis

*NODS* National Opinion Research Center DSM Screen for Gambling Problems, *SOGS* South Oaks Gambling Screen, *DSM* Diagnostic and Statistical Manual of Mental Disorders, *PGSI* Problem Gambling Severity Index, *MADRS* The Montgomery-Asberg Depression Rating Scale, *GD* Gambling Disorder, *GA* Gamblers Anonymous, *SSRIs* Selective serotonin reuptake inhibitors

### Non-pharmacological therapies

Detailed information about each treatment, as well as the control group and the outcome measures, is described in Table 3.

**Table 3:** Summary table of non-pharmacological treatments for gambling disorder and brief results of the included studies

Article	Therapy (ies)	Control	Outcome (Primary measures)	Results
Nilsson et al. 2019 [35]	10 sessions of BCT	10 sessions of CBT	NODS; TLFB-G	BCT group had statistically significant improvements on every outcome; there was not, however, a significant difference between BCT and CBT.
Penna et al. 2018 [28]	16 sessions of an Exercise program	Stretching session	GFS-SR; psychiatric comorbidities	Both groups had statistically significant improvements on both outcomes. The exercise group had significantly greater improvements on psychiatric comorbidities compared to control, but no significantly differences on GFS-SR scale.
Casey et al. 2017 [25]	6 sessions of I-CBT	I-MFS and waitlist	G-SAS; SOGS; GRCS; GUS; DASS; gambling amount; gambling frequency^a^	Compared to the waitlist, the I-CBT group had significant reductions on every outcome, at follow-up. Compared to the I-MFS group, I-CBT showed significant reductions in gambling urges (GUS), gambling related cognitions (GRCS) and in depression, anxiety and stress (DASS).
Bouchard et al. 2017 [20]	4 VR sessions in CBT	4 imagination control stimuli sessions in CBT	CPGI; DIG; GRCS	The VR + CBT group had significant reductions on every outcome, at post treatment. However, there was no significant differences compared to the control group.
Smith et al. 2015 [47]	Twelve 1 h sessions of ET	Twelve 1 h sessions of CT	VGS	ET group significantly improved on VGS score, at post-treatment and at follow-up. However, there was not a significant difference between the treatment and the control groups.
Lee et al. 2014 [18]	12 weekly sessions of CCT	Brief check-in phone calls	G-SAS; BSI; DAS; STIC	CCT group significantly improved on gambling symptoms (G-SAS) and mental distress (BSI), compared with control group, at post-treatment and follow-up. Compared with control, the CCT group significantly improved on systemic functioning (STIC) at post treatment, but did not show significant differences at follow-up. There was no difference between groups on DAS.
Grant et al. 2011 [38]	6 h sessions over 8 weeks of ID+MI	Gamblers Anonymous	PG-YBOCS	ID+MI group significantly improved on PG-YBOCS score, compared to the GA group, at post-treatment. This significant improvement was maintained at the follow-up.
Myrseth et al. 2011 [31]	Eight weekly 50 min sessions for 8 weeks CBT	Escitalopram	G-SAS; PGVAC	At post-treatment (8 weeks) and at 6 months follow-up, both groups showed improvements on every outcome. However, there was no significantly difference between groups.
Marceaux et al. 2010 [48]	Two weekly sessions over 8 weeks of CBT-mapping or TSF	Waitlist	DSM-IV criteria; self-efficacy; frequency of gambling^a^; desire to gamble	At post treatment and 6 months follow up, both treatment groups significantly improved on every outcome, except for desire to gamble. However, there was no significant differences between both treatment groups.
Grant et al. 2009 [39]	Six 1 h session for 8 weeks of ID+MI	Gamblers Anonymous	PG-YBOCS; G-SAS	ID+MI group significantly improved on every outcome, after the 8 weeks treatment, compared to the GA group.
Myrseth et al. 2009 [29]	6 sessions of 2 h CBT in group	Waitlist	Money spent per week; GINC and DSM-IV	CBT group had a significant decrease in DSM-IV criteria, compared to control; however, the improvements on money spent per week and GINC were non significant, compared to control. The CBT group significantly improved on every outcome, at 3-months follow-up.
Carlbring et al. 2009 [36]	four 50 min sessions of MI or eight 3 h sessions of CBT	Waitlist	NODS	Both CBT and MI groups significantly improved on NODS, compared to the control group, at post-treatment and at 12 months follow-up. There were no significant differences between the two active treatments at any time.
Cunningham et al. 2009 [21]	E-mailed PFI	Waitlist	CPGI; Money spent per 3 months; largest money gamble in a day in the past 3 months	PFI group significantly reduced the total amount of money spent, at follow up, compared with control; there were also improvements on the maximum amount of money spent on one occasion and gambling severity (CPGI) at follow-up, but with no significant difference compared with control.
Carlbring et al. 2008 [37]	8-Week I-CBT	Waitlist	NODS; anxiety; depression; quality of life	I-CBT group significantly improved on every outcome, compared with control; the improvements were maintained significant at 6-, 18- and 36-month follow-up.
Dowling et al. 2007 [16]	Twelve 2 h sessions of group CBT or twelve 1.5 h sessions of individual CBT	Waitlist	Gambling frequency^a^ and duration; money inserted; expenditure; BDI-II scores; STAI scores; CSEI scores	The individual group, compared to control, significantly improved on every outcome. The group format treatment, compared to control, significantly improved on every outcome, except for STAI state anxiety scores and CSEI scores. Compared to each other, the two intervention groups showed no significant differences at post-treatment; However, after the 6 month follow-up period, 92% of the individual treatment group participants no longer had criteria for pathological gambling, compared with only 60% of the group treatment group participants.
Petry et al. 2006 [41]	8 Weeks of a CBT workbook or eight 1 h sessions of CBT	Gamblers Anonymous	SOGS; days spent gambling; money spent gambling; abstinence	At post-treatment and 12-month follow-up, the individual CBT group significantly improved on gambling severity (SOGS) and on money spent gambling, compared to the control and to the CBT workbook group. CBT group had significantly greater abstinence rates at post-treatment, compared with the other groups. The outcome days spent gambling did not register any differences between groups.
Melville et al. 2004 [14]	2 weekly 90 min node-link-mapping-enhanced CBT group for 8 weeks	Waitlist	DSM-IV; self-ratings of control of gambling; refrain from gambling; desire to gamble; BDI; BAI	The mapping group significantly improved on every outcome at post-treatment, compared to control. Regarding depression (BDI) and anxiety (BAI), the mapping group had significant reductions compared to the control group, but only the depression improvements were maintained at 6-month follow-up.
Ladouceur et al. 2003 [49]	120 min weekly sessions of CT in group for 10 weeks	Waitlist	DSM-IV; perceived self-efficacy; gamblers’ perception of control; desire to gamble and frequency of gambling^a^	CT group, at post-treatment and compared to control, significantly improved on every outcome, except for frequency of gambling and desire to gamble. Analysis of data from 6-, 12- and 24-month follow-ups revealed maintenance of therapeutic gains.
Ladouceur et al. 2001 [50]	Weekly 60 min individual CT session for 20 weeks	Waitlist	SOGS; DSM-IV; gamblers’ perception of control; frequency of gambling^a^; perceived self-efficacy; and desire to gamble	CT group significantly improved on every outcome measure, compared with control group; analysis of data from 6 and 12-month follow-up revealed maintenance of therapeutic gains.
Sylvain et al. 1997 [51]	1 or 2 weekly 60-90 min CBT sessions to a maximum of 30 h of treatment	Waitlist	SOGS; perception of control; frequency of gambling^a^; perceived self-efficacy; desire to gamble; DSM-III-R	CBT group significantly improved, compared with control group, on every outcome measure, except for hours spent gambling; analysis of data from 6 and 12-month follow-up revealed maintenance of therapeutic gains.
Echeburua et al. 1996 [6]	6 h of CT or 6.5 h of ET or 12.5 h of CT + ET	Waitlist	< 3 episodes of gambling during follow up	ET group and CT group significantly improved on every outcome, compared to the combined treatment and to control, at 6 months follow-up. At 12 months follow up, the ET group already had a significant difference compared to CT.
McConaghy et al. 1991 [52]	Five 20 min sessions of ID	Aversive therapy, imaginal relaxation, exposure therapy	Cessation or controlled gambling symptoms	At follow-up, 79% of the patients who received ID therapy showed significant improvements on cessation/controlled gambling symptoms, compared with only 53% of the patients of the control group.

*BCT* Behavioural couples therapy, *CBT* Cognitive-behavioural therapy, *I-CBT* Internet-based cognitive-behavioural therapy, *VR* Virtual Reality, *CT* Cognitive therapy, *ET* Exposure therapy, *CCT* Congruence couples therapy, *ID* Imaginal desensitization, *MET* Motivational Enhancement Therapy, *TSF* Twelve-step facilitated, *PFI* Personalized feedback intervention, *I-MFS* Internet-based monitoring, feedback and support, *NODS* National Opinion Research Center DSM Screen for Gambling Problems, *TLFB-G* The gambling timeline followback, *GFS-SR* Gambling Follow-up Scale self-report version, *G-SAS* The gambling symptom assessment scale, *SOGS* South Oaks Gambling Screen, *GUS* Gambling Urge Scale, *DASS* Depression Anxiety Stress Scale, *CPGI* The Canadian Problem Gambling Index, *DIG* Diagnostic Interview for Gambling, *GRCS* Gambling Related Cognitions Scale, *VGS* Victorian Gambling Screen, *BSI* Brief Symptom Inventory, *DAS* Dyadic Adjustment Scale, *STIC* Systemic Therapy Inventory of Change, *PG-YBOCS* Pathological Gambling – Yale Brown Obsessive-Compulsive Scale, *PGVAC* Pathological Gambling Visual Analogue Craving Scale, *DSM* Diagnostic and Statistical Manual of Mental Disorders, *GINC* Gamblers Inventory of Negative Consequences, *BDI* Beck Depression Inventory, *STAI* State-Trait Anxiety Inventory, *CSEI* Coopersmith Self-Esteem Inventory, *BAI* Beck Anxiety Inventory

^a^ a) number of gambling sessions, b) number of hours spent gambling and c) total amount of money they had spent on gambling during the previous week

#### Cognitive-behaviour therapy (CBT)

Nine studies using this therapy were included in this review. Three studies assessed the group format, while four studies evaluated the individual format. In Myrseth et al. [29], 14 patients were randomized to either a short-term (6 sessions of 2 h) group CBT session or to a waitlist control, and, at post-treatment, the authors reported significant reductions on DSM-IV criteria met for treatment patients, compared to control. At 3-month follow-up the CBT group showed significant improvements on GINC scale, DSM-IV criteria met and gambling behaviour. Carlbring et al. [36] also tested a group CBT treatment (3 h sessions per week, for 8 weeks) compared to a waitlist control group; the results at post-treatment and follow-up showed significant reductions on the outcome measure (NODS score), compared to controls. In Myrseth’s et al. [31] controlled trial, 30 participants were randomized to either an individual CBT treatment (50 min/weekly sessions for 8 weeks) or a control doing an escitalopram treatment; the results at post-treatment and 6-month follow-up showed improvements on gambling severity (G-SAS score) and craving (PGVAC score) for both groups, without a significant difference between them. Petry et al. [41] also tested individual CBT therapy (1 h/weekly sessions for 8 weeks), by randomizing 231 patients to 3 groups: an individual CBT group, a self-CBT workbook or a gamblers anonymous referral control group. To eliminate possible confounders, both active treatments were also referred to gamblers anonymous. At post-treatment and 12-month follow-up, the individual CBT group had significantly greater improvements on abstinence, gambling severity (SOGS score) and money spent gambling, compared to the other groups; there was no difference between groups in days spent gambling. Sylvain’s et al. [51] randomized 29 patients to either an individual CBT group (1 or 2 sessions per week), or to a waitlist control; at post-treatment and follow-up, the authors reported significant improvements on gambling severity, perception of control, self-efficacy and desire to gamble, compared to the controls; while there was no change in gambling behaviour. Dowling et al. [16] randomized 56 female patients in 3 groups: two CBT groups delivered in a group format (12 sessions of 2 h) or an individual format (12 sessions of 1.5 h) and a waitlist control group. The results at post-treatment and follow-up showed significant improvements for the individual treatment on all gambling behaviour and psychological functioning measures (BDI-II scores, STAI-trait anxiety standard scores and Coopersmith SEI scores), compared to controls; while the group treatment did not improve in STAI-state anxiety scores and CSEI scores, compared to controls. After the 6-month follow-up period, 92% of the individual CBT group participants no longer had criteria for GD, compared with only 60% of the group CBT group participants.

Three studies explored innovative formats of CBT. Casey et al. [25] randomized 174 patients to either an internet-based CBT program (6 sessions), a monitoring, feedback, and support group or a waitlist control. The results at post-treatment and follow-up showed significant improvements for the internet-based CBT program group on gambling behaviour, gambling severity (GSAS and SOGS scores), gambling urge (GUS score), gambling related cognitions (GRCS score), depression, anxiety and stress (DASS score), when compared to controls, and in gambling urge, gambling related cognitions and stress compared to the monitoring, feedback, and support group. Carlbring’s et al. [37] randomized 66 patients to either 8 weeks of internet-based CBT or waitlist control, and the results at post-treatment and follow-up showed significant improvements on gambling severity (NODS score), anxiety, depression and quality of life, compared with controls. Bouchard’s et al. [20] trial evaluated the efficacy of virtual reality sessions of gambling scenarios in CBT, compared to a control consisting of sessions with no exposure. The virtual reality sessions induce gambling cravings in the patients, by exposing them to gambling scenarios and cues [20]. The results showed that the virtual reality group had significant reductions on gambling severity (CPGI score), diagnostic criteria (DIG score) and dysfunctional gambling beliefs (GRCS score) at 2-week follow-up, however, without statistically significant differences compared to controls.

#### Cognitive therapy (CT)

Two studies assessed the efficacy of this therapy’s group format and one study addressed the individual format. Ladouceur’s et al. [50] randomized 101 patients into an individual CT group (twenty 60 min sessions) or into a waitlist control group. The results at post-treatment and follow-up showed that the treatment group had significant improvements on gambling severity, desire to gamble, perception of control and self-efficacy, compared to controls. In a similar study, Ladouceur et al. [49], tested a group format CT (ten 2 h sessions), compared to a waitlist control. This study found similar results at post-treatment evaluation; in the follow-up, although still significant, the improvements tended to diminish over time. Echeburua’s et al. [6] controlled trial randomized 64 participants into 4 groups: 6 h of CT, 6.5 h of ET, 12.5 h of combination of CT + ET, and a waitlist control group. At 6 and 12-month follow-up, the CT group showed a significant improvement on gambling frequency compared to the combined treatment and to controls.

#### Exposure therapy (ET)

Two studies addressed this therapy’s efficacy. Echeburua’s et al. [6] randomized 64 participants into the 4 groups already described. At follow-up, the ET group showed significant improvements on gambling frequency compared to the combined treatment and to controls. At 12 months follow-up assessment, the ET group results were significantly better compared to the CT group. In Smith’s et al. [47], 99 participants were randomized to either 12 individual ET sessions or 12 CT sessions. The results at post-treatment and 9 months follow-up showed a significant reduction on VGS scores within the ET group, but no statistically significant difference compared to the CT group.

#### Imaginal desensitization and motivational interviewing

Two studies where these therapies were combined were included in this review, as well as two studies where each was performed individually. Carlbring et al. [36] randomized 150 patients into a motivational interviewing group, a CBT group and a waitlist control group. The results showed a significant improvement of gambling severity, compared to the control group, at post-treatment and follow-up. However, there was no significant difference when compared to CBT at any time. In McConaghy et al. [52], 120 patients were randomized to either an imaginal desensitization therapy group or to a control. At follow-up, a significantly higher proportion of patients who received imaginal desensitization showed cessation or controlled gambling symptoms, when compared to controls. In Grant’s et al. [39], 68 patients were randomized to either an imaginal desensitization plus motivational interviewing group or a gamblers anonymous referral control group. The results at post-treatment showed significantly greater acute improvements on behaviour and urge symptoms (PG-YBOCS score) and gambling severity (G-SAS score), compared to controls. In Grant et al. [38], a similar trial was done, with the results being assessed at 6 months follow-up and showing a significant improvement on reducing the PG-YBOCS score for the treatment group, compared to controls.

#### Couples therapy

Two studies addressing this therapy were included in this review. Nilsson’s et al. [35] randomized 136 patients and respective partners to either a behavioural couples therapy group (10 sessions over 12 weeks) or a CBT control group. Both treatment and control group significantly reduced gambling severity, compared to baseline; however, there were no significant differences between groups. The only difference, although not significant, was a greater adherence to treatment for the patient, compared to control. Lee’s et al. [18] randomized 16 participants into a couples therapy group or a control group, receiving only counselling. The results at post-treatment and follow-up showed significant improvements on gambling symptoms (G-SAS score) and mental distress (BSI score), compared to controls; systemic functioning (STIC score) only had significant improvements at post treatment; also, no significant differences in couple relationship (DAS score) were found between treatment and control couples at post-treatment and follow-up.

#### Other therapies

The node-link mapping-enhanced therapy is a technique that enhances the gambler and therapist communication [53]. It consists in a visual representation technique with the goal of detecting interrelations between thoughts, emotions, actions, and environmental influences [53]. It uses maps containing boxes (nodes) and lines (links) to visually illustrate thoughts, feelings or information [53]. It can be used in association with other therapies, for example, with CBT [53]. In Melville’s et al. [14] controlled trial, 19 patients were randomized to either a node-link mapping enhanced therapy or a waitlist control group. The mapping group met significantly fewer DSM-IV diagnostic criteria at post-treatment than the control group. The self-rated ability to control gambling and the ability to refrain from gambling increased for both groups, but the size of the increase was significantly larger for the mapping group. Desire to gamble decreased significantly from pre- to post-treatment for the mapping group, contrarily to the control group, that increased. At 6-month follow-up, the desire to gamble had no significant change. Regarding depression and anxiety (BDI and BAI scores), the mapping group had significant reductions compared to the control group, but only the depression improvements were maintained at 6-month follow-up.

Twelve-step facilitated treatment is a group therapy similar to gamblers anonymous, that approaches cognitive, emotional, behavioural, social, and spiritual areas [48]. It is widely used for the treatment of alcohol dependence, so, its efficacy was also tested in GD [48]. Marceaux’s et al. [48] controlled trial randomized 49 patients to a waitlist control group and 2 therapy groups: a node-link mapping-enhanced cognitive-behavioural group and a twelve-step facilitated treatment. Both therapies consisted in 2 weekly sessions of 90 min over 8 weeks. At post-treatment, both treatment groups significantly decreased on DSM-IV criteria met, increased on self-efficacy and decreased on frequency of gambling. However, there was no significant change on desire to gamble over time nor significant differences between both treatment groups. These effects were maintained at 6-month follow-up. In Cunningham’s et al. [21] controlled trial, 49 patients were randomized to a mailed personalized feedback intervention group and to a waitlist control group. This treatment consisted in a report with the number and types of gambling activities compared to the general population of the same age and sex. It also reports the patients CPGI score and gives them a list of techniques and advices to stop gambling. The results showed that the study group significantly reduced the total amount of money spent, at 3 months follow-up, compared with controls. The personalized feedback intervention group also reduced the maximum amount of money spent on one occasion and gambling severity (CPGI score) at follow-up, but with no significant differences compared with controls. Physical activity has already proven to be efficient in the treatment of other disorders, such as alcohol and drug dependence [28]. It helps reduce stress levels, cravings and improves the mood [28]. Penna’s et al. [28] controlled trial randomized 59 patients into a group doing an exercise program (16 sessions for 8 weeks) and a control group doing stretching-only sessions. This physical activity sessions consisted of 10-min stretching plus 40-min running at 70–85% of the estimated maximum heart rate for age, while the control group had 50 min sessions of only stretching exercises. The results showed that GD patients from the exercise program improved on gambling severity, psychiatric comorbidity and gambling craving. However, at follow-up, only the decrease of psychiatric comorbidities was statistically significant, when compared to the control group.

## Discussion

The main objective of the present review was to synthesise and compare the efficacy and durability of treatment effects of various non-pharmacological therapies for GD. The general outcome in the included studies was improvements on gambling symptoms, severity and/or behaviour, assessed by the scales and scores mentioned, and by measures like gambling frequency, money spent gambling or time spent gambling. Nine of the included studies (40.91%) achieved the outcomes with statistical significance and confirmed the efficacy of the treatments tested [6, 14, 25, 36–39, 50–52]; nine other studies (40.91%) confirmed the efficacy of the treatments tested, but some of the outcomes did not reach significance [16, 18, 21, 28, 29, 41, 48, 49]; and 4 of the included studies (18.18%) were not able to confirm the efficacy of the therapies, because the outcomes were non-significant [20, 31, 35, 47]. Nonetheless, every study obtained results in favour of the therapies, despite some of them not being statistically significant. Some therapies, although having proved its efficacy, did not reach significance, because their trial designs did not include a waitlist group, only active treatment groups.

Considering the trials that addressed CBT, the evidence confirmed this treatment’s efficacy, as seven studies registered significant improvements on the majority of the outcomes. Both the individual and the group approach to this therapy had positive results; however, in Dowling et al. [16], the two formats were compared and the individual one proved the be superior at follow-up, with a lower percentage of people diagnosed with GD. Nonetheless, group treatment appears to have some potential benefits, as it utilizes group cohesion and social support to restore the patients’ self-respect and self-efficacy, and allows the patients to learn from other patients’ successes and failures, by promoting observational learning [29]. It is also more time-efficient for the therapist and has better cost-benefits [29]. Two studies tested an internet-based approach for CBT and the results were satisfactory, proving to have a similar efficacy as a face-to-face CBT treatment [25, 37]. The internet-based approach is an innovative format of CBT as it can be offered as a way of enabling gamblers to overcome many of the barriers that prevent them from accessing traditional forms of treatment, as it is more versatile and convenient to the patient than the classical therapy [25]. It increases treatment options, allowing more patients to be treated, maintaining the efficacy of a face-to-face CBT [25]. Other study [20] assessed the possibility of integrating virtual reality exposure sessions to the CBT treatment. It is also an innovative format because, by inducing gambling cravings and desires in the patient, it allows them to practice the CBT techniques safely in the therapist’s office [20]. The study consisted of a pilot trial conducted on a small population and, despite not having achieved significance, this study provided a solid empirical basis, justifying a trial at a larger scale. Longer duration treatments did not appear to be more beneficial than shorter duration ones, as every study that specified the duration had significant improvements on the outcomes. Lastly, the results also supported the short- and long-term efficacy of this therapy, as the improvements remained significant regardless of the duration of the follow-up, that varied from 3 months to 3 years.

This review included 3 studies evaluating CT. Both group [6, 49] and individual [50] formats demonstrated their efficacy, yet, the individual format improved significantly more outcome measures, favouring this format of CT. This therapy revealed to be effective at both moderate and long-term, by maintaining or improving the therapeutic effects at 6-, 12- and 24-months follow-up. ET proved to be effective in one study [6], and when compared to CT at-follow-up, also showed to be superior. Although both therapies showed its efficacy separately, surprisingly, when they were combined (ET + CT) [6], the treatment did not lead to significant improvements. Another study [47] did not find differences between ET and CT, but the authors justified the results with the high drop-out rate of the participants in the trial.

Imaginal desensitization and motivational interviewing also had positive results, with 2 studies [29, 36] proving its efficacy separately, and another 2 studies proving these therapies to be effective when combined [38, 39]. The improvements were maintained at follow-up, being this a valid long-term therapy. When compared to CBT, motivational interviewing therapy revealed to be four times less time-consuming and a lot cheaper [36].

Couples therapy only showed significant improvements on some outcome measures, when compared to a waitlist control [18]. When compared to a standard CBT treatment, the results did not show that involving the patient’s partner in the therapy led to better results [35]. It was only reported a better adherence to treatment, being that this therapy’s biggest strength [35]. Low adherence is one of the biggest obstacles of this disorder’s treatment, alongside with the low rates of treatment-seeking patients (2.6%), as data from gambling helplines have reported [2].

Of the alternative therapies explored through the included studies, the node-link mapping therapy [14, 48] and the twelve-step facilitated treatment [48] proved to be effective, while the personalized feedback intervention [21] did not reach significance on most outcomes at follow-up, and the exercise program [28] only presented significant benefits on reducing gambling comorbidities, failing to reach significant improvements on gambling severity.

## Strengths and limitations

The limitations of this review are:

1. The inclusion of studies whose control group is an active treatment. These trials only give information about the efficacy of one therapy compared to another; it would be advantageous if they included a waitlist control group;
2. The bibliographical searches were only performed at PubMed and Cochrane Library databases, when it would have been beneficial to have searched other databases;
3. The heterogeneity of rating instruments used as outcome measures, that made some comparisons difficult;
4. The duration, formats and sample sizes of some therapies were sometimes discrepant. Also, some articles also used small samples, lacked confidence limits or did not blind the study personnel to treatment.
5. The exclusion of patients with other comorbidities, given that the clinical reality is that the majority of these patients in fact have comorbidities that should be addressed in therapy.

This review’s strengths consist in:

1. The inclusion of patients diagnosed with GD. We decided to exclude all controlled trials who included “problem gamblers” or “participants worried about their gambling behaviour” but were not diagnosed with GD;
2. The inclusion of randomized controlled trials only makes the results trustworthy;
3. A great part of the included trials was published in the past decade, what makes the results in this review updated;
4. An adequate background about GD treatment (pharmacological and non-pharmacological) was given, mentioning the main conclusions of previous reviews on this subject.

## Conclusions

Several psychotherapies included in this review show promising results in the treatment of GD. However, there is a need for additional trials to further confirm the benefits of each therapy, as some of them have been tested in only one trial yet. Further studies should consider testing other therapies than CBT, and some innovative formats, such as the internet-based format, given its advantages. Once these therapies have been studied sufficiently in the treatment of GD and there is a reasonable empirical basis, a gold-standard therapy can be defined. Further studies should also consider different therapies for GD patients that range in severity. Additionally, an updated meta-analysis on this topic is needed.

## Data Availability

The datasets used and/or analysed during the current study are available from the corresponding author on reasonable request.

## Abbreviations

BAI: Beck Anxiety Inventory
BDI: Beck Depression Inventory
BSI: Brief Symptom Inventory
CASP: Critical Appraisal Skills Programme
CBT: Cognitive-behavioural Therapy
CPGI: The Canadian Problem Gambling Index
CSEI: Coopersmith Self-Esteem Inventory
CT: Cognitive therapy
DAS: Dyadic Adjustment Scale
DASS: Depression Anxiety Stress Scale
DIG: Diagnostic Interview for Gambling
DSM: Diagnostic and Statistical Manual of Mental Disorders
ET: Exposure Therapy
GD: Gambling Disorder
GFS-SR: Gambling Follow-up Scale self-report version
GINC: Gamblers Inventory of Negative Consequences
GRCS: Gambling Related Cognitions Scale
G-SAS: The gambling symptom assessment scale
GUS: Gambling Urge Scale
MADRS: The Montgomery-Asberg Depression Rating Scale
NODS: National Opinion Research Center DSM Screen for Gambling Problems
PGSI: Problem Gambling Severity Index
PGVAC: Pathological Gambling Visual Analogue Craving Scale
PG-YBOCS: Pathological Gambling – Yale Brown Obsessive-Compulsive Scale
SOGS: South Oaks Gambling Screen
SSRI: Selective serotonin reuptake inhibitors
STAI: State-Trait Anxiety Inventory
STIC: Systemic Therapy Inventory of Change
TLFB-G: The gambling timeline followback
VGS: Victorian Gambling Screen
